# A new species of *Bathyeliasona* (Annelida, Polynoidae) from the abyssal plain of the eastern Indian Ocean

**DOI:** 10.3897/zookeys.1293.208455

**Published:** 2026-09-25

**Authors:** Cheah Hoay Chuar, Iffah Iesa, Tashfia Raquib

**Affiliations:** 1 St. John’s Island National Marine Laboratory, Tropical Marine Science Institute, National University of Singapore, 18 Kent Ridge Road, 119227, Singapore, Singapore St. John’s Island National Marine Laboratory, Tropical Marine Science Institute, National University of Singapore Singapore Singapore; 2 Lee Kong Chian Natural History Museum, National University of Singapore, 2 Conservatory Drive, 117377, Singapore, Singapore Lee Kong Chian Natural History Museum, National University of Singapore Singapore Singapore

**Keywords:** Christmas Island Seamount Province, deep sea, ROV, scale worm, taxonomy

## Abstract

A new species of deep-sea polynoid, *Bathyeliasona oceanxplorer***sp. nov**., is described from the abyssal plain located in international waters between Christmas Island and the Cocos (Keeling) Islands in the eastern Indian Ocean from a single specimen collected at a depth of 4977 m. The new species is characterised using morphology and molecular data from four genetic markers (COI, 16S rRNA, 18S rRNA, and 28S rRNA). *Bathyeliasona oceanxplorer***sp. nov**. is distinguished from its congeners by a combination of characters, including the possession of 18 segments, slender frontal filaments, the presence of a buccal bulb, and ventral pygidial lobes with weakly undulate margins. This is the first species of *Bathyeliasona* for which the elytra are described and illustrated. This finding highlights the great value of ROV-based sampling for recovering comparatively intact deep-sea polynoid specimen with most delicate features preserved.

## Introduction

Knowledge of deep-water benthic fauna in the eastern Indian Ocean remains limited. Prior to 2020, no systematic deep-water benthic sampling had been conducted in waters around Christmas Island and the Cocos (Keeling) Islands, Western Australia (Murray et al. 2025; Kupriyanova et al. 2026). Two biodiversity surveys were recently carried out in 2021 and 2022 in the waters off Australia’s Indian Ocean Territories (IOT) (O’Hara 2024). Kupriyanova et al. (2024) provided a catalogue of the annelid morphospecies collected during these surveys. Using three collection methods (beam trawl, Gandalf net, and Sherman sled), approximately 867 specimens representing 34 families were obtained. However, several annelid families that are typically abundant and diverse in benthic samples were absent or only poorly represented, including cirratulids, dorvilleids, and spionids, likely because of their small body size (Kupriyanova et al. 2024). A similar pattern was reported by Chuar et al. (2021) in a study of deep-water annelids, collected from depths of up to 1796 m off southwest Java in the eastern Indian Ocean using beam trawls and dredges.

Polynoid scale worms were among the better represented taxa in the IOT collections. Twelve morphospecies were recognised, including a *Bathyeliasona* morphospecies likely allied with *Bathyeliasona nigra* (Hartman, 1967) from depths of 3780–4990 m (Kupriyanova et al. 2024). In the most recent study treating annelid material from the IOT, three new species of Polynoidae, a potential new polynoid genus, and one known species of *Anotochaetonoe* Britayev & Martin, 2006 were reported and described (Kupriyanova et al. 2026).

The genus *Bathyeliasona* Pettibone, 1976 currently comprises four valid species (Read and Fauchald 2026), mostly with wide geographical distributions and broad bathymetric ranges. The four species were originally described from widely separated deep-sea regions: *Bathyeliasona abyssicola* (Fauvel, 1913) from the Gulf of Gascony (Bay of Biscay) in the northeastern Atlantic at 4380 m; *B. kirkegaardi* (Uschakov, 1971) from the Aleutian Trench in the North Pacific at 7180 m; *B. mariaae* Bonifácio & Menot, 2018 from the Clarion-Clipperton Zone in the eastern Pacific at 4328–4823 m; and *B. nigra* (Hartman, 1967) from the South Sandwich Islands in the South Atlantic at 2553–2575 m. Subsequent records have expanded the known distributions of three species: *B. abyssicola* was later reported from the Aleutian Trench (Levenstein 1961), *B. kirkegaardi* from the Kermadec Trench, Sunda Trench, and the northeastern Atlantic near Portugal (Kirkegaard 1956; Hartmann-Schröder 1975; Pettibone 1976), and *B. nigra* from southwest of Sri Lanka in the Indian Ocean (Kirkegaard 1995) as well as from southern and eastern Australia (Murray et al. 2025). In contrast, *B. mariaae* is currently known only from the eastern Pacific nodule fields (Bonifácio and Menot 2018). Gonzalez et al. (2025) summarised the recorded depth ranges of all valid *Bathyeliasona* species and observed that *B. abyssicola* has the deepest record within the genus, at 7974 m, and is also the most frequently recorded polynoid species from depths of 3000 m or greater.

**Figure 1.**
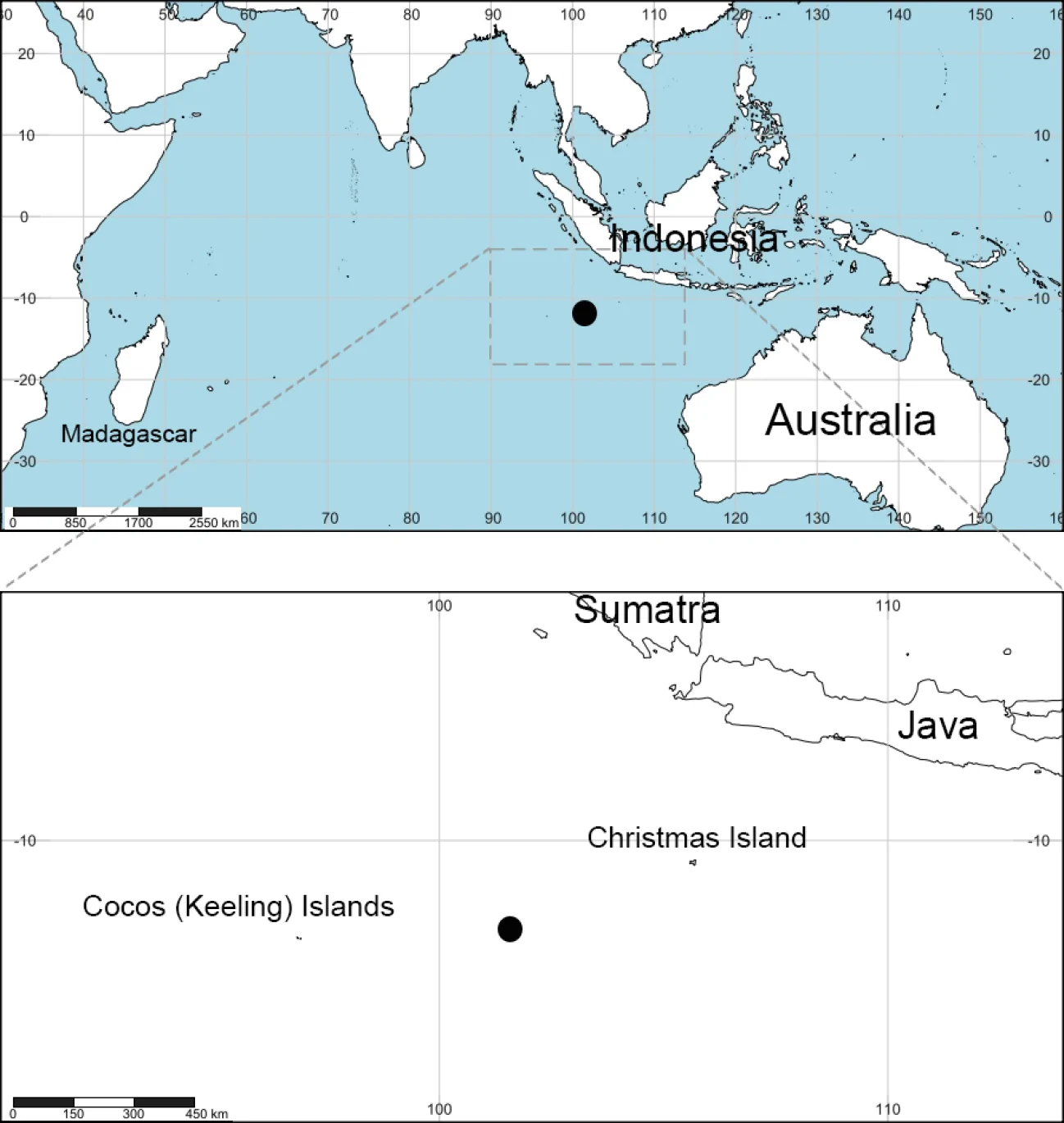
Sampling locality of *Bathyeliasona oceanxplorer* sp. nov. in the eastern Indian Ocean, indicated by a black circle. The map was created using SimpleMappr and modified in Adobe Photoshop 27.4.0.

**Figure 2.**
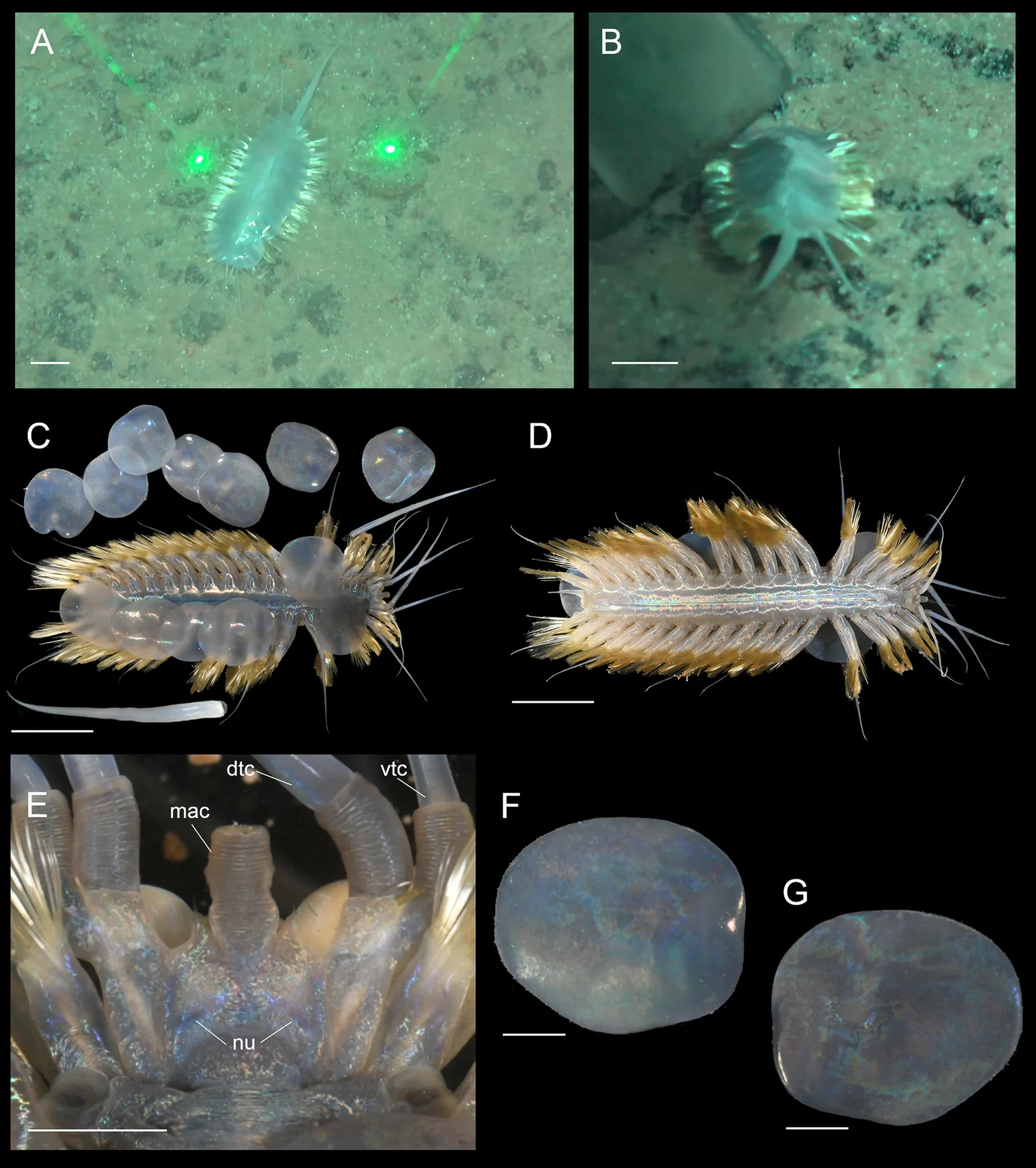
Live specimen of *Bathyeliasona oceanxplorer* sp. nov. **A**. *In situ* image, distance between laser points 10 cm; **B**. *In situ* image just before collection using the ROV suction sampler; **C**. Whole body with some elytra detached, dorsal view; **D**. Whole body, ventral view; **E**. Anterior end, dorsal view; **F**. Elytron, dorsal view; **G**. Elytron, ventral view. Abbreviations: dtc = dorsal tentacular cirrus, mac = median antenna ceratophore, nu = nuchal organs, vtc = ventral tentacular cirrus. Scale bars: 2 cm (**A–D**); 3 mm (**E**); 5 mm (**F, G**).

Following the ratification of the Agreement on the Conservation and Sustainable Use of Marine Biological Diversity of Areas beyond National Jurisdiction (BBNJ, or High Seas Agreement) in September 2025 (United Nations 2025), the National University of Singapore and OceanX jointly conducted a deep-sea biodiversity expedition in October 2025 to international waters between Christmas Island and the Cocos (Keeling) Islands (Fig. 1). A range of sampling equipment was deployed, including a remotely operated vehicle (ROV), free-fall benthic lander, multinet plankton sampler, large volume water transfer system, and box corer, enabling the collection of biological material across a broad size range from pelagic and benthic environments. Among the specimens collected across seven ROV dives was a single large polynoid scale worm from the abyssal plain, estimated to be ~ 10 cm long based on ROV camera observations (Fig. 2A). The animal was conspicuous on an otherwise sparsely populated seafloor and was collected with the ROV suction sampler (Suppl. material 1).

In this paper, we describe a new species of *Bathyeliasona*, increasing the number of valid species in the genus from four to five. We provide a morphological description together with molecular data (cytochrome c oxidase subunit I [COI], 16S rRNA, 18S rRNA, 28S rRNA), using phylogenetic analyses to support species delineation rather than to resolve broader relationships among genera. This is the first species of *Bathyeliasona* for which the elytra are described and figured. In previously described species, elytral morphology could not be documented because the elytra are readily shed and are often lost during collection (Pettibone 1976; Bonifácio and Menot 2018; Murray et al. 2025). This also highlights the value of ROV- or submersible-based sampling which often yield more intact specimens than using bottom trawling or dredging. The absence of key diagnostic features, particularly the elytra and chaetae in damaged specimens is problematic for polynoid taxonomy (Kupriyanova et al. 2026).

## Materials and methods

### Sample collection

A single specimen (ZRC.ANN.2366) was collected from an abyssal plain between Christmas Island and the Cocos (Keeling) Islands during the National University of Singapore–OceanX (NUS–OX) Expedition to the eastern Indian Ocean in October 2025 (Fig. 1). A total of seven ROV dives were conducted during the cruise. The specimen was collected using the suction sampler of the ROV*Chimaera* deployed from the R/V OceanXplorer (Fig. 2B; Suppl. material 1).

### Morphological examination

The live specimen was photographed onboard using a Nikon D850 DSLR camera with 60 mm macro lens. The specimen was then fixed and preserved in 75% ethanol, with one elytron preserved in absolute ethanol for subsequent molecular analyses. The preserved specimen was examined using a Nikon SMZ25 stereomicroscope. Body length was measured from the median antenna ceratophore to the posterior border of the last segment, and body width was measured at the widest segment, including chaetae. Additional photographs were taken using a Leica M205 C stereomicroscope (Leica DMC5400 camera), a Nikon SMZ25 stereomicroscope (Nikon Digital Sight 10 camera), and a Nikon D850 DSLR camera with 60 mm macro lens. Focus-stacked images from the Leica M205 C and Nikon SMZ25 were processed using the Leica Focus Stacking module in Leica Application Suite X and the Extended Depth of Focus (EDF) module in Nikon NIS-Elements AR v6.20.00, respectively. DSLR images were stacked using Adobe Photoshop 26.11.

For scanning electron microscopy (SEM), chaetae were mounted on brass stubs, air-dried, sputter-coated with platinum using a JEOL JEC-3000FC Auto Fine Coater, and examined with a JEOL JSM-6010PLUS/LV SEM.

All examined material, including dissected parts and SEM stubs, is deposited in the Zoological Reference Collection (**ZRC**) of the Lee Kong Chian Natural History Museum, National University of Singapore, Singapore, under the same catalogue number as the type material.

### DNA extraction, amplification, and sequencing

DNA was extracted from an elytron using the DNeasy Blood & Tissue Kit (Qiagen), following the manufacturer’s protocol, with a final elution volume of 50 µL. PCR amplifications were carried out on a ProFlex 3 × 32-well PCR System (Applied Biosystems), targeting two mitochondrial markers (COI, 16S rRNA) and two nuclear markers (18S rRNA, 28S rRNA).

A fragment of ~ 650 bp of COI was amplified using polyLCO (5’-GAYTATWTTCAACAAATCATAAAGATATTGG-3’) and polyHCO (5’-TAMACTTCWGGGTGACCAAARAATCA-3’) (Carr et al. 2011) under the following conditions: 95 °C for 4 min; 40 cycles of 94 °C for 30 s, 52 °C for 1 min, and 72 °C for 75 s; followed by a final extension at 72 °C for 8 min.

A fragment of ~ 450 bp of 16S rRNA was amplified using Ann16SF (5’-GCGGTATCCTGACCGTRCWAAGGTA-3’) (Sjölin et al. 2005) and 16SbrH (5’-CCGGTCTGAACTCAGATCACGT-3’) (Palumbi 1996) using the following profile: 95 °C for 4 min; 35 cycles of 94 °C for 30 s, 52 °C for 1 min, and 72 °C for 75 s; followed by a final extension at 72 °C for 8 min.

A fragment of ~ 1800 bp of 18S rRNA was amplified using 18SA (5’-AYCTGGTTGATCCTGCCAGT-3’) (Medlin et al. 1988) and 18SB (5’-ACCTTGTTACGACTTTTACTTCCTC-3’) (Nygren and Sundberg 2003) under the following conditions: 95 °C for 4 min; 35 cycles of 94 °C for 30 s, 52 °C for 1 min, and 72 °C for 3 min; followed by a final extension at 72 °C for 8 min.

A fragment of ~ 800 bp of 28S rRNA was amplified using 28SC1’ (5’-ACCCGCTGAATTTAAGCAT-3’) and 28SD2 (5’-TCCGTGTTTCAAGACGG-3’) (Lê et al. 1993) using the following profile: 96 °C for 1 min; 29 cycles of 95 °C for 30 s, 62 °C for 1 min, and 72 °C for 1 min; followed by a final extension at 72 °C for 7 min.

Each PCR reaction had a total volume of 25 µL and contained 5 µL template DNA, 2.5 µL of each primer (10 µM), 12.5 µL GoTaq G2 Green Master Mix (Promega), and nuclease-free water to volume. PCR products were checked on a 1% agarose gel stained with SYBR Safe (Invitrogen) in 1× TAE buffer for 30 min and visualised using a SafeBlue Illuminator System (Major Science). Successful amplicons were purified using the QIAquick PCR Purification Kit (Qiagen), following the manufacturer’s instructions. Purified products were Sanger-sequenced using BigDye Terminator v3.1 chemistry (Applied Biosystems) on an ABI PRISM 3730xl Genetic Analyzer (Applied Biosystems) by 1^st^ BASE (Singapore), using the same primers as in the PCR reactions.

### Phylogenetic analyses

The phylogenetic placement of the new species within Macellicephalinae was assessed using maximum-likelihood (ML), based primarily on the dataset compiled by Murray et al. (2025), with additional reference sequences from Neal et al. (2026) (Table 1). Raw trace files for each gene of the new species were assembled into consensus sequences using the *de novo* assembly function in Geneious Prime 2026.1.2. Reference sequences were obtained from GenBank (NCBI; Sayers et al. 2025) (Table 1). For each gene, sequences were aligned independently using MAFFT v. 7.490 (Katoh and Standley 2013) implemented in Geneious Prime, with the E-INS-i strategy. Each alignment was visually inspected and cropped to the target gene region. Ambiguously aligned positions were removed using trimAl v. 1.5.1 with the gappyout setting (Capella-Gutiérrez et al. 2009). The four trimmed alignments were then concatenated in Geneious Prime.

**Table 1.** GenBank accession numbers used in the phylogenetic analyses. Sequences of *Bathyeliasona oceanxplorer* sp. nov. generated in this study are shown in bold.

**Taxon**	**GenBank Accession Number**				**Reference**
	**COI**	**16S rRNA**	**18S rRNA**	**28S rRNA**	
*Abyssarya acus* Bonifácio & Menot, 2018	MH233279	MH233182	MH233232	—	Bonifácio and Menot (2018)
*Bathyeliasona mariaae* Bonifácio & Menot, 2018	MH233260	MH233197	MH233243	—	Bonifácio and Menot (2018)
*Bathyeliasona nigra* (Hartman, 1967)	PQ360818	PQ368174	PX048982	PQ368243	Murray et al. (2025)
***Bathyeliasona oceanxplorer* sp. nov**.	**PZ946871**	**PZ946646**	**PZ946647**	**PZ946648**	**This study**
*Bathyfauvelia glacigena* Bonifácio & Menot, 2018	MH233272	MH233186	MH233236	—	Bonifácio and Menot (2018)
*Bathyfauvelia ignigena* Bonifácio & Menot, 2018	MH233264	MH233202	—	—	Bonifácio and Menot (2018)
*Bathykurila guaymasensis* Pettibone, 1989	MH233265	MH233203	MH233248	—	Bonifácio and Menot (2018)
*Bathypolaria magnicirrata* (Neal, Barnich, Wiklund & Glover, 2012)	—	PQ368175	PX048983	PQ368242	Murray et al. (2025)
*Bathypolaria* sp. 173	MH233281	MH233151	MH233206	—	Bonifácio and Menot (2018)
*Bathypolaria* sp. 608	MH233268	MH233175	MH233227	—	Bonifácio and Menot (2018)
*Branchipolynoe pettiboneae* Miura & Hashimoto, 1991	KY753825	KY753825	KY753840	KY753840	Zhang et al. (2018)
*Branchipolynoe trifurcus* (Miura & Desbruyères, 1995)	MW646933	MW654558	MW654529	—	Hatch et al. (2020)
*Branchipolynoe segonzaci* (Miura & Desbruyères, 1995)	MK357906	MK211418	MK211412	—	Wu et al. (2019)
*Bruunilla magnantennata* Murray, Burghardt, Gunton, Nizar, Nikolic & Wilson, 2025	—	PQ368177	—	—	Murray et al. (2025)
*Bruunilla nealae* Bonifácio & Menot, 2018	—	MH233158	MH233216	—	Bonifácio and Menot (2018)
*Bruunilla posteroantennata* Murray, Burghardt, Gunton, Nizar, Nikolic & Wilson, 2025	—	PQ368178	—	—	Murray et al. (2025)
*Bruunilla* sp. 692	MH233263	MH233201	MH233247	—	Bonifácio and Menot (2018)
*Cladopolynoe sandersi* (Pettibone, 1985)	JN852923	JN852889	JN852821	JN852851	Norlinder et al. (2012)
*Hodor anduril* Bonifácio & Menot, 2018	MH233258	MH233190	MH233239	—	Bonifácio and Menot (2018)
*Hodor hodor* Bonifácio & Menot, 2018	MH233257	MH233189	MH233238	—	Bonifácio and Menot (2018)
*Laetmonice murrayae* Flaxman & Kupriyanova, 2024 (outgroup)	PQ360847	PQ368210	PX056280	PQ368247	Murray et al. (2025)
*Macellicephala brenesorum* Neal, Brasier & Wiklund, 2018	MG905047	MG905035	MG905041	—	Neal et al. (2018)
*Macellicephala clarionensis* Bonifácio & Menot, 2018	MH233270	MH233184	MH233234	—	Bonifácio and Menot (2018)
*Macellicephala gloveri* Neal, Brasier & Wiklund, 2018	KX867447	KX867371	MG905042	—	Neal et al. (2018)
*Macellicephala irisae* Neal, Wiklund, Smith, Benn, Kemp, Dahlgren & Glover, 2026	PZ310488	PZ318984	PZ314305	—	Neal et al. (2026)
*Macellicephala linseae* Neal, Brasier & Wiklund, 2018	KX867448	KX867378	MG905043	—	Neal et al. (2018)
*Macellicephala cf. macintoshi*	PQ360851	PQ368214	PX049008	PQ368246	Murray et al. (2025)
*Macellicephala monroi* Neal, Brasier & Wiklund, 2018	—	MG905036	MG905044	—	Neal et al. (2018)
*Macellicephala parvafauces* Bonifácio & Menot, 2018	MH233275	MH233173	MH233225	—	Bonifácio and Menot (2018)
*Macellicephala patersoni* Neal, Brasier & Wiklund, 2018	—	MG905037	MG905045	—	Neal et al. (2018)
*Macellicephala violacea* (Levinsen, 1886)	JX119016	MG905038	MG905046	—	COI: Shields et al. (2013); 16S, 18S: Neal et al. (2018)
Macellicephalinae sp.	—	PQ368215	PX049009	PQ368249	Murray et al. (2025)
*Ovatocirra flavida* Chuar, Hookabe, Fujiwara, Iesa, Kobayashi, Zhang & Jimi, 2026	PZ812644	PZ827277	PZ827616	—	Chuar et al. (2026)
*Ovatocirra shinkaiae* Chuar, Hookabe, Fujiwara, Iesa, Kobayashi, Zhang & Jimi, 2026	PZ812648	PZ827281	PZ827619	—	Chuar et al. (2026)
*Peinaleopolynoe sillardi* Desbruyères & Laubier, 1988	MH115414	MH127421	—	—	Hatch et al. (2020)
*Polaruschakov investigatoris* Murray, Burghardt, Gunton, Nizar, Nikolic & Wilson, 2025	PQ360853	PQ368216	—	—	Murray et al. (2025)
*Polaruschakov lamellae* Bonifácio & Menot, 2018	MH233250	MH233150	MH233205	—	Bonifácio and Menot (2018)
*Polaruschakov omnesae* Bonifácio & Menot, 2018	MH233254	MH233164	—	—	Bonifácio and Menot (2018)
*Themis undomarginata* (Zhang, Chen & Qiu, 2018)	MG799385	MG799376	MG799379	MG799381	Zhang et al. (2018)
*Yodanoe desbruyeresi* Bonifácio & Menot, 2018	MH233251	MH233156	—	—	Bonifácio and Menot (2018)
*Yodanoe* sp. 659-3	MH233273	MH233195	—	—	Bonifácio and Menot (2018)

Maximum likelihood analysis was performed using IQ-TREE 3.0.1 (Wong et al. 2026) on the CIPRES Science Gateway (Miller et al. 2010). A partitioned analysis was conducted in IQ-TREE following the partition model framework of Chernomor et al. (2016). The best-fit substitution model for each partition was selected using ModelFinder (Kalyaanamoorthy et al. 2017). The selected models were GTR+F+I+G4 (COI), TIM2+F+I+G4 (16S rRNA), TN+I+R2 (18S rRNA), and TIM2+F+G4 (28S rRNA). Branch support was assessed using 1000 ultrafast bootstrap (UFBoot2) replicates (Hoang et al. 2018) and 1000 SH-aLRT replicates (Guindon et al. 2010). The tree was rooted with the aphroditid *Laetmonice murrayae* Flaxman & Kupriyanova, 2024, visualised and annotated using iTOL v. 7 (Letunic and Bork 2024), and further edited in Adobe Illustrator 30.4. All sequences generated in this study were deposited in GenBank (NCBI) and are listed in Table 1.

A pairwise genetic distance matrix for the COI sequences was generated in MEGA 12.1.2 (Kumar et al. 2024) using uncorrected p-distance. Gaps or missing data were treated using pairwise deletion.

## Systematics

### Family Polynoidae Kinberg, 1856

**Subfamily Macellicephalinae Hartmann-Schröder, 1971**

**Genus *Bathyeliasona* Pettibone, 1976**

#### Bathyeliasona oceanxplorer sp. nov.

Taxon classificationAnimaliaPhyllodocidaPolynoidae

67E08243-4926-5853-8452-F012DC513449

https://zoobank.org/ADE1EF58-4DAF-47ED-AEAE-455E093F9DA5

**Figure 3.**
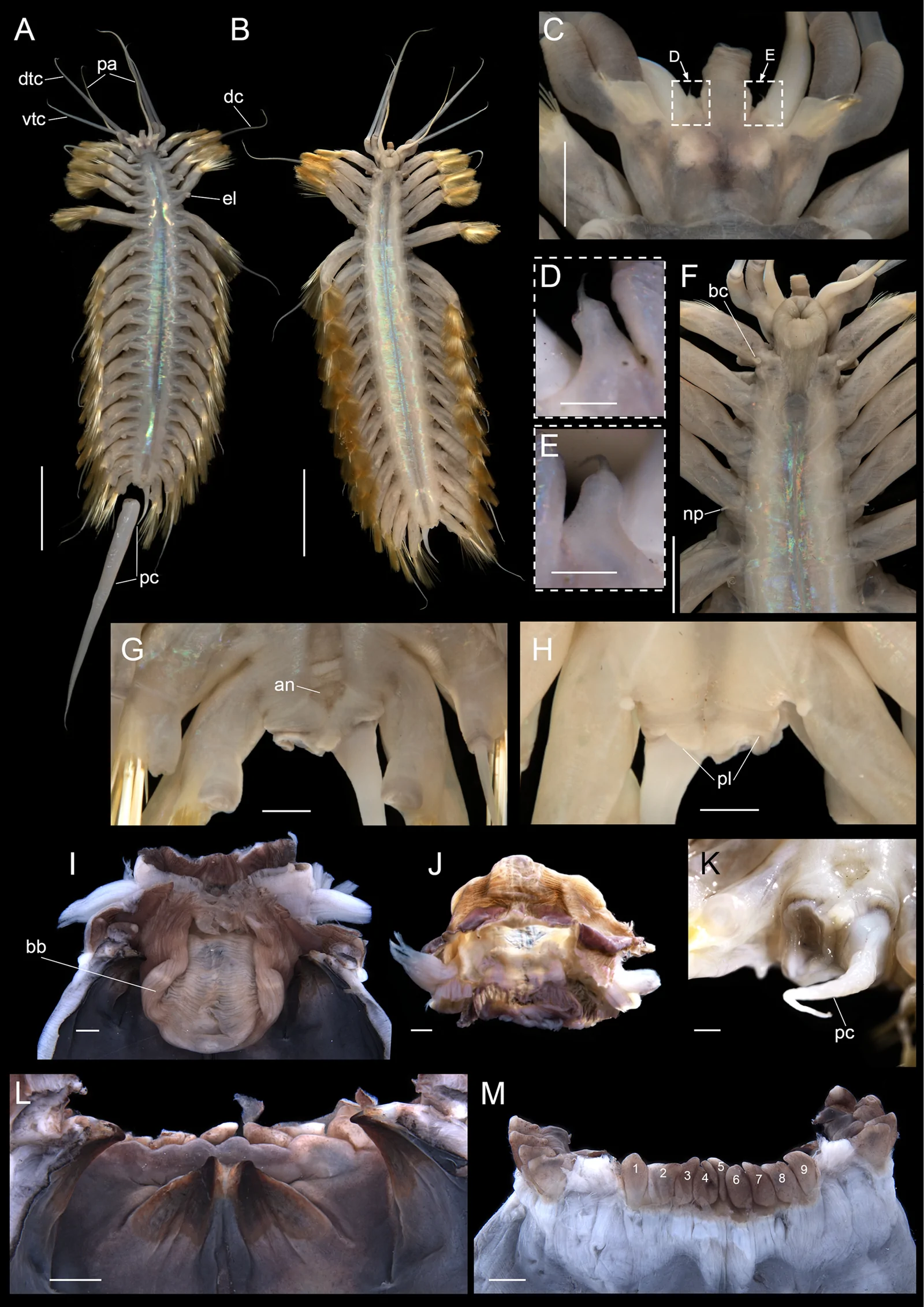
Preserved specimen of *Bathyeliasona oceanxplorer* sp. nov. **A**. Whole body, dorsal view; **B**. Whole body, ventral view; **C**. Anterior end, dorsal view; **D**. Frontal filament (left); **E**. Frontal filament (right); **F**. Anterior end, ventral view; **G**. Posterior end, dorsal view; **H**. Posterior end, ventral view; **I**. Pharynx dissected, showing the buccal bulb at distal end; **J**. Buccal bulb removed from the pharynx and turned inside out; **K**. Pygidial end, posterior view; **L**. Jaws; **M**. Pharyngeal papillae. Abbreviations: an = anus, bb = buccal bulb, bc = buccal cirrus, dc = dorsal cirrus, dtc = dorsal tentacular cirrus, el = elytrophore, np = nephridial papilla, pa = palp, pc = pygidial cirrus, pl = pygidial lobe, vtc = ventral tentacular cirrus. Scale bars: 2 cm (**A, B**); 3 mm (**C**); 500 µm (**D, E**); 5 mm (**F**); 2 mm (**G, H**); 1 mm (**I–M**).

**Figure 4.**
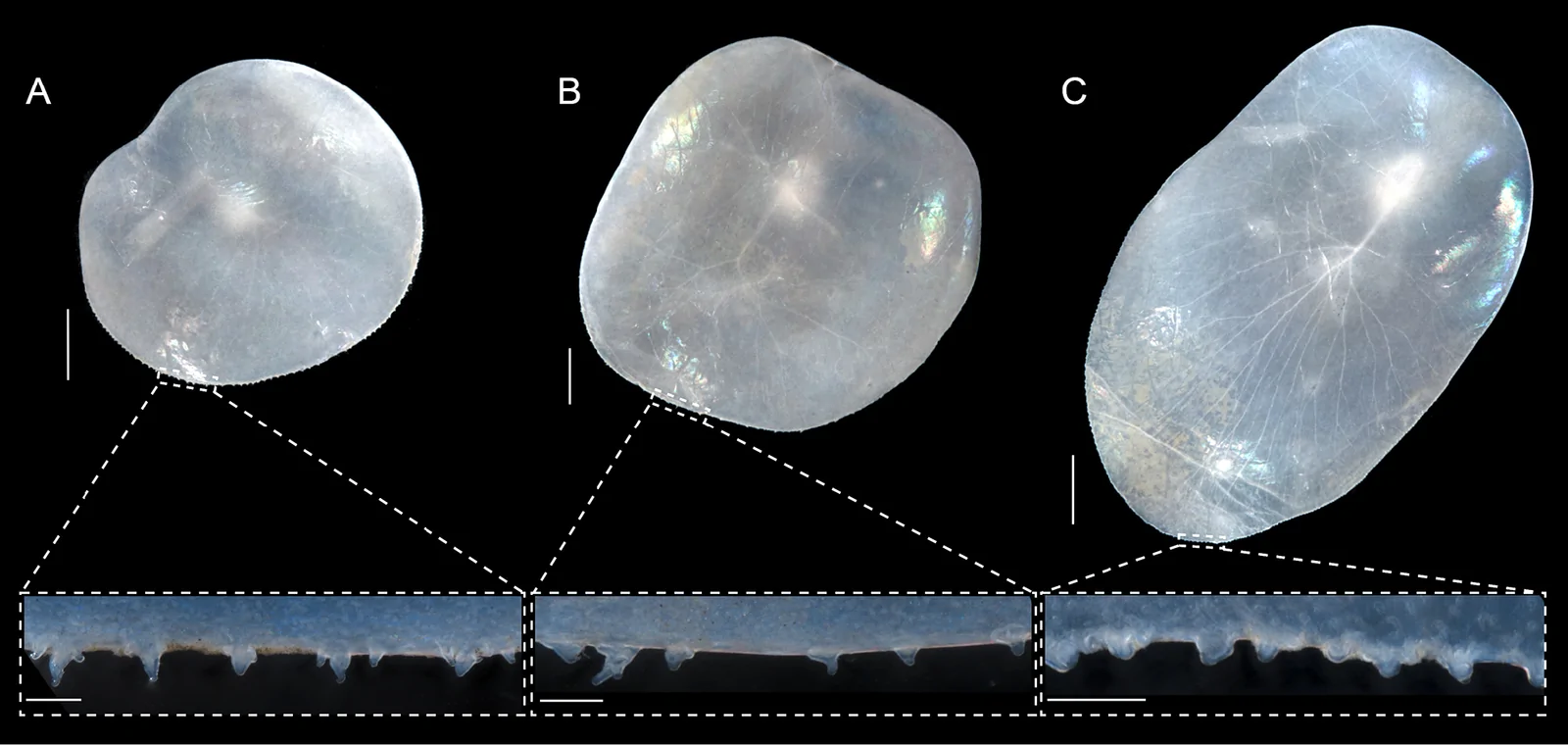
Preserved elytra of *Bathyeliasona oceanxplorer* sp. nov., each with an inset showing a close-up of the marginal papillae. **A**. Elytron from an anterior segment; **B**. Elytron from a mid-body segment; **C**. Elytron from a posterior segment. Scale bars: 3 mm (**A–C**); 200 µm (**insets A–C**).

**Figure 5.**
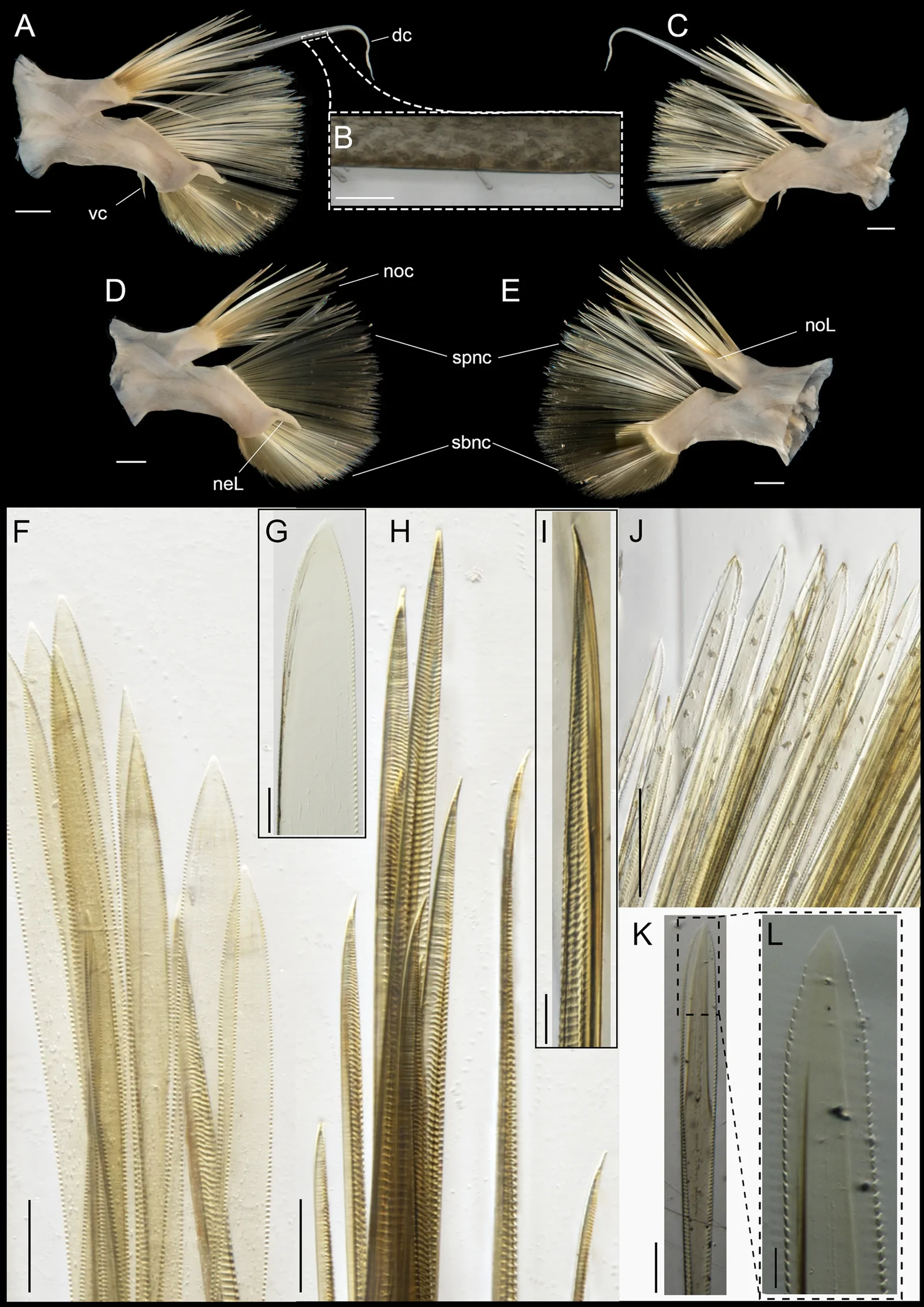
Parapodia and chaetae of *Bathyeliasona oceanxplorer* sp. nov. **A**. Left cirrigerous parapodium from segment 10, anterior view; **B**. Papillae on a dorsal cirrus; **C**. Left cirrigerous parapodium from segment 10, posterior view; **D**. Left elytrigerous parapodium from segment 11, anterior view; **E**. Left elytrigerous parapodium from segment 11, posterior view; **F**. Supraacicular neurochaetae; **G**. Distal end of a supraacicular neurochaeta; **H**. Notochaetae; **I**. Distal end of a notochaeta; **J**. Subacicular neurochaetae; **K**. Subacicular neurochaeta; **L**. Distal end of a subacicular neurochaeta. Abbreviations: dc = dorsal cirrus, noc = notochaetae, noL = notopodial acicular lobe, neL = neuropodial acicular lobe, sbnc = subacicular neurochaetae, spnc = supraacicular neurochaetae, vc = ventral cirrus. Scale bars: 2 mm (**A, C, D, E**); 200 µm (**B, G, I, K**); 500 µm (**F, H, J**); 50 µm (**L**).

**Figure 6.**
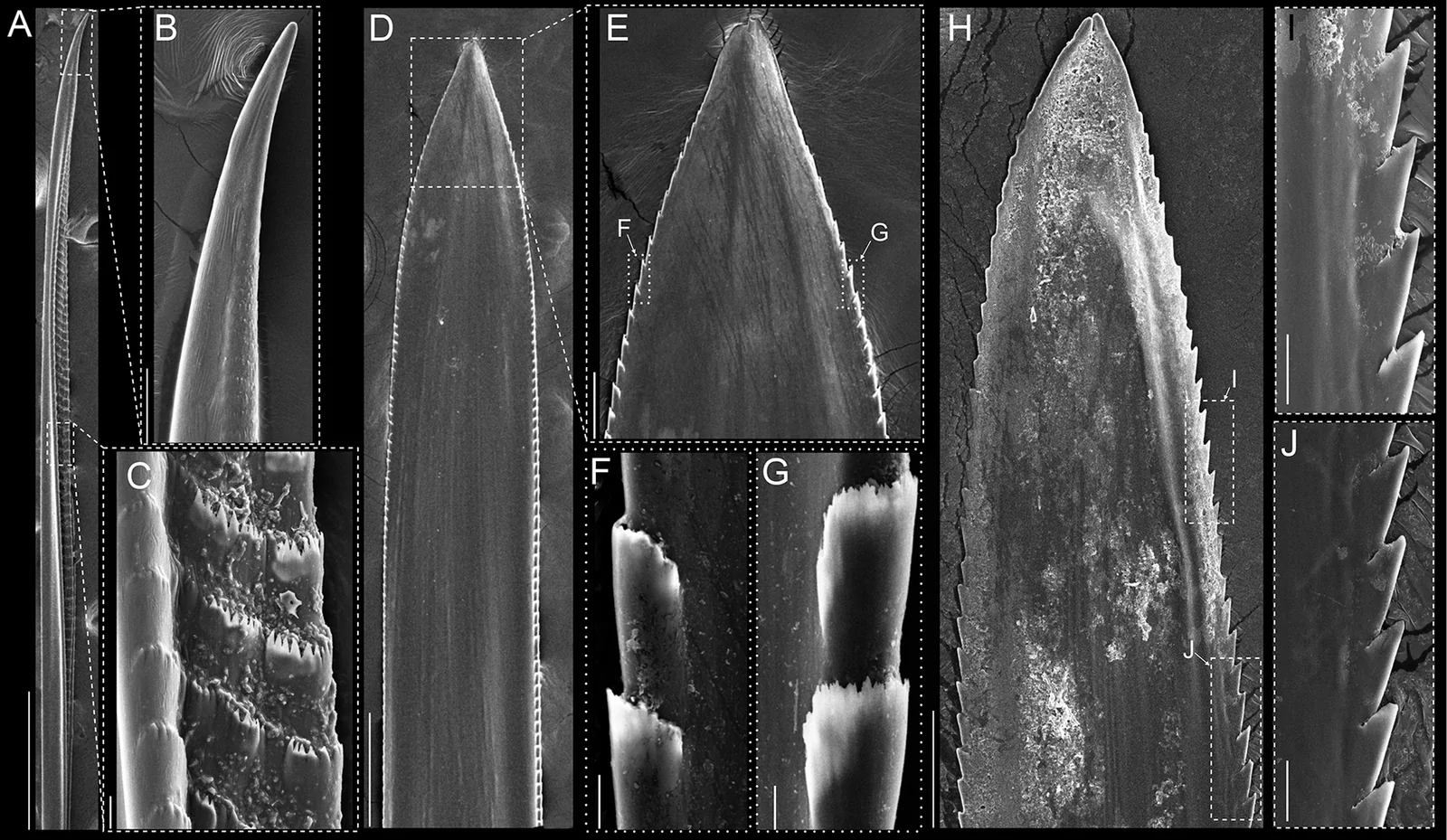
SEM micrographs of the chaetae of *Bathyeliasona oceanxplorer* sp. nov. **A**. Notochaeta; **B**. Distal end of notochaeta; **C**. Spinous rows on the mid notochaetal blade; **D**. Supraacicular neurochaeta; **E**. Distal end of supraacicular neurochaeta; **F, G**. Close-up views of the serrated lateral margins; **H**. Subacicular neurochaeta; **I, J**. Close-up views of the serrated lateral margins. Scale bars: 500 µm (**A**); 50 µm (**B, E, H**); 10 µm (**C, I, J**); 200 µm (**D**); 5 µm (**F, G**).

**Figure 7.**
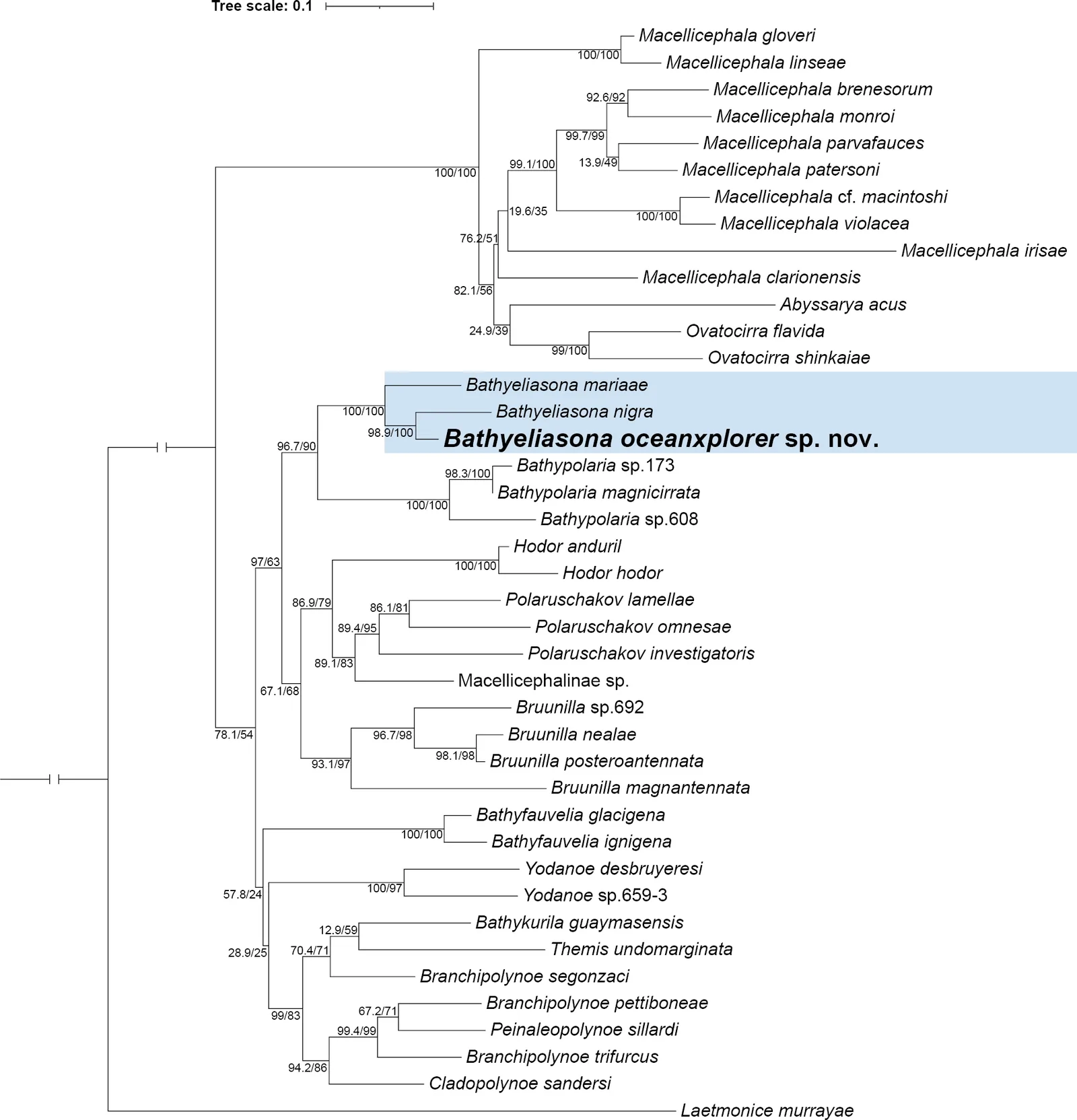
Maximum-likelihood phylogeny of Macellicephalinae inferred from the concatenated four-gene dataset (COI, 16S rRNA, 18S rRNA, and 28S rRNA). The *Bathyeliasona* clade is highlighted in blue, and the new species is shown in bold. *Laetmonice murrayae* was used as the outgroup. Node labels indicate SH-aLRT and ultrafast bootstrap support values based on 1000 replicates.

Figs 2, 3, 4, 5, 6, 7; Tables 1, 2

##### Material examined.

***Holotype***. Indian Ocean • Abyssal plain between Christmas Island and the Cocos (Keeling) Islands, Station CHR23, Dive CHR0597; −11.9829, 101.5860; depth 4977 m; 18 Oct. 2025; Suction sampler, ROV*Chimaera* operated from R/V OceanXplorer, NUS–OX Expedition (OXR20251007); GenBank: PZ946646–PZ946648, PZ946871; ZRC.ANN.2366.

##### Diagnosis.

Body with 18 segments; prostomial lobes anteriorly rounded, each with a slender, short frontal filament inserted anteriorly; eight pairs of elytra, with short, non-uniformly sized conical papillae along the posterior half of the margins; subacicular neurochaetae with distinct central rib on the distal half; nephridial papillae present from segment 5 onwards, all small; buccal bulb present; ventral pygidial lobes with weakly undulate distal margins.

##### Description.

Body 85 mm long, 48 mm wide, with 18 segments; robust, compact, dorsoventrally flattened. Dorsum of live specimen covered by large, overlapping, thick, semi-transparent elytra (Fig. 2A–C). Body surface smooth, iridescent *in vivo* (Fig. 2C–E); preserved specimen greyish in ethanol (Fig. 3A). With eight pairs of elytra on segments 2, 4, 5, 7, 9, 11, 13, and 15, attached to prominent, bulbous elytrophores, completely covering the dorsum (Figs 2A–C, 3A). Elytra oval to subreniform, thick, with short, non-uniformly sized conical marginal papillae along the posterior half of the margin (Figs 2C, 2F, 2G, 4A–C); iridescent *in vivo* when illuminated (Fig. 2C, F, G), opaque and greyish when preserved (Fig. 4). Cirrigerous segments with prominent cylindrical dorsal cirrophores, inserted on posterior side of notopodia (Fig. 5C); cirrostyle tapering, sparsely papillate, extending beyond tips of neurochaetae (Fig. 5A, B).

Prostomium bilobed; lobes pronounced, anteriorly rounded, with slender, short frontal filaments inserted anteriorly (Figs 2E, 3C–E). Eyes absent. A pair of nuchal organ pits dorsally on the posterior part of prostomial lobes, visible in live specimen, indiscernible after preservation (Figs 2E, 3C). Median antenna with ceratophore long and prominent, inserted in median notch, style lost (Figs 2E, 3C). Lateral antennae absent. Facial tubercle absent. Palps smooth, tapering, long, reaching segment 6 (Figs 2C, 2D, 3A, 3B).

Tentaculophores with chaetae (Figs 2E, 3C). Tentacular styles distally tapering, reaching segment 6, sparsely papillated, papillae small; dorsal style slightly longer than ventral (Figs 2C, 2D, 3A, 3B).

Pharynx long, dark grey, reaching segment 11, with nine pairs of subtriangular, equal-sized distal pharyngeal papillae (Fig. 3M); two pairs of jaws, both with smooth margins (Fig. 3L). An inverted buccal bulb at distal end of pharynx (Fig. 3I, J).

Parapodia biramous, notopodia shorter than neuropodia (Fig. 5A, C–E). Notopodia cylindrical; acicular lobe distally elongated, tapering (Fig. 5E). Neuropodia large, subrectangular; acicular lobe distally elongated, digitiform (Fig. 5A, D); post-chaetal lobe truncate (Fig. 5C, E). Notochaetae ~ 50 per ramus, stouter than neurochaetae, distally straight to slightly curved, with numerous spinous rows on convex side, tips pointed and smooth (Figs 5H, 5I, 6A–C). Neurochaetae numerous, distally greatly expanded and flattened, with serrated lateral margins (Figs 5F, 5G, 5J–L, 6D–J); supraacicular neurochaetae ~ 2× as wide as subacicular neurochaetae (Fig. 5F, G, J–L); subacicular neurochaetae with central rib distally (Fig. 5J–L). Parapodia of last segment similar to preceding segments.

Buccal cirri inserted basally on neuropodia of segment 2, smooth, tapering, slightly longer than subsequent ventral cirri (Fig. 3B, F). Ventral cirri on segments 3–18 short, inserted subdistally on neuropodia (Figs 3B, 5A).

Nephridial papillae present from segments 5–18, small, digitiform to subconical (Fig. 3B, F); a rudimentary nephridial papilla on right parapodium of segment 4.

Pygidium terminal, with dorsal anus (Fig. 3G); a pair of ventral pygidial lobes, distal margins weakly undulate (Fig. 3H). Pygidial cirri inserted in a subovate depression, style tapering, sparsely papillate, papillae minute (Fig. 3A, K). Left cirrus 60 mm long, much thicker and ~ 6× longer than right cirrus, probably regenerating; detached during collection and retained in the same container (Fig. 3A).

##### Phylogeny.

In the concatenated phylogeny based on COI, 16S rRNA, 18S rRNA, and 28S rRNA, *Bathyeliasona oceanxplorer* sp. nov. was recovered within a well-supported *Bathyeliasona* clade (100/100; Fig. 7), where it was resolved as sister to *B. nigra* (98.9/100), with *B. mariaae* sister to this clade. Of the four valid species of *Bathyeliasona*, sequence data are available only for *B. nigra* and *B. mariaae*. Uncorrected pairwise COI p-distances between *Bathyeliasona oceanxplorer* sp. nov. and these two congeners were 17.7% and 18.6%, respectively. These results confirm the placement of the new species within *Bathyeliasona* and indicate that it is genetically well differentiated from its sequenced congeners.

##### Etymology.

The specific epithet *oceanxplorer* is used for the research vessel OceanXplorer, in recognition of its support for the expedition during which the type material was collected. The name is treated as a noun in apposition.

##### Distribution.

Eastern Indian Ocean, on the abyssal plain between Christmas Island and the Cocos (Keeling) Islands (Fig. 1).

##### Ecology.

Collected from the abyssal plain seafloor (4977 m) at water temperature of 1.2 °C.

##### Behaviour.

When first observed through the ROV camera, the polynoid was stationary on the seafloor and showed no obvious movement, even when the ROV landed nearby. It attempted to move away only when the suction sampler approached and began drawing it in (Suppl. material 1).

##### Remarks.

*Bathyeliasona oceanxplorer* sp. nov. resembles *B. abyssicola* (Fauvel, 1913) and *B. nigra* (Hartman, 1967) in having 18 body segments (Table 2). However, the frontal filaments of both *B. abyssicola* and *B. nigra* are subulate, whereas those of *B. oceanxplorer* sp. nov. are slender and short (Fig. 3D, E). In this character, the new species more closely resembles *B. kirkegaardi* (Uschakov, 1971), as both species have anteriorly rounded prostomial lobes with slender frontal filaments. Even so, *B. oceanxplorer* sp. nov. differs from *B. kirkegaardi* in having 18 (instead of 17) segments, the presence of a buccal bulb (not reported in the latter), and a different pygidial lobe shape (Table 2). The new species also resembles *B. mariaae* in having slender frontal filaments, the presence of nuchal organs, and subacicular neurochaetae with central rib, but differs in several aspects: the frontal filaments are shorter, reaching only ~ 1/2 the length of the median antenna ceratophore (Fig. 3C), rather than reaching its distal end; the nuchal organs are situated near the posterior part of the prostomial lobes (Fig. 2E), rather than on the mid-anterior part; and nephridial papillae are present (Fig. 3F), rather than absent.

**Table 2.** Comparison of morphological characters between *Bathyeliasona oceanxplorer* sp. nov. and its congeners.

**Character/Species**	***Bathyeliasona abyssicola* (Fauvel, 1913)**	***Bathyeliasona kirkegaardi* (Uschakov, 1971)**	***Bathyeliasona mariaae* Bonifácio & Menot, 2018**	***Bathyeliasona nigra* (Hartman, 1967)**	***Bathyeliasona oceanxplorer* sp. nov**.
No. of segments	18	17	17	18	18
Buccal bulb	Not reported	Not reported	Not reported	Not reported	Present
Frontal filaments shape	Subulate	Slender, filiform	Subulate, slender, long (reaching distal end of median antenna ceratophore)	Subulate	Slender, short (reaching 1/2 length of median antenna ceratophore)
Nuchal organ	Not reported	Not reported	Present	Not reported	Present
Nephridial papillae	All small	All small	Absent	Enlarged on segments 10–12	All small
Subacicular neurochaetae central rib	Not reported	Not reported	Yes	Not reported	Yes
Pygidial lobe	Subtriangular	Subconical	Subconical	Subconical	Ventral margin weakly undulate
Reference	Pettibone (1976)	Pettibone (1976)	Bonifácio and Menot (2018)	Pettibone (1976); Murray et al. (2025)	This study

## Discussion

*Bathyeliasona oceanxplorer* sp. nov. is the first species of the genus described from the eastern Indian Ocean, adding to the sparse but growing knowledge of abyssal annelids in this region (Kupriyanova et al. 2026). As some *Bathyeliasona* species have wide recorded geographical distributions, as noted in the Introduction, the new species is unlikely to be restricted to this region. Increased sampling effort will therefore be important for establishing its true distribution, which is likely to be underestimated owing to the limited sampling effort at abyssal depths.

All described species of *Bathyeliasona* show a high degree of overall morphological resemblance, particularly in the shape of the parapodia and the general pattern of serrations on the noto- and neurochaetae. This close resemblance among species, combined with the limited and often damaged material available, means that few characters are currently reliable for distinguishing them. It is therefore useful to distinguish characters that appear more promising for species discrimination from those that remain uncertain because of specimen condition.

Several potentially informative characters cannot yet be evaluated across the genus because the relevant features are often damaged, lost, or not described in the available literature. Nuchal organs, reported in the present study, are not yet reliable as diagnostic characters. Even in the new species, the nuchal organ depressions were clearly visible only in fresh material (Fig. 2E) and were indiscernible after preservation (Fig. 3C). Elytral morphology is similarly limited; although the shape of the elytra and the presence of marginal papillae are described here for *B. oceanxplorer* sp. nov., the elytral morphology of the currently valid species remains unknown, because the elytra detach easily and were not retained with previous material (Pettibone 1976; Bonifácio and Menot 2018; Murray et al. 2025). The central rib on the subacicular neurochaetae, which gives them a bilimbate appearance (Fig. 5J–L), is a comparable case. Although Bonifácio and Menot (2018) reported this feature in some specimens of *B. mariaae*, neurochaetae are frequently lost or damaged in preserved collections of *Bathyeliasona* (Pettibone 1976). While we include the presence of central rib on neurochaetae in the diagnosis of the new species, it cannot yet be assessed with high confidence across the genus, as it remains unclear whether this feature is genuinely absent in other species or has simply gone unobserved because of specimen condition. We regard it as a potentially informative feature that requires examination across additional, well-preserved specimens of *Bathyeliasona* species before its diagnostic value can be established.

Recent studies of *Bathyeliasona* have used the relative stoutness of the notochaetae and neurochaetae as a character in the generic diagnosis (Bonifácio and Menot 2018; Murray et al. 2025). This character traces back to Pettibone (1976), who stated: “Notosetae stout, spinous, more slender than neurosetae”. Bonifácio and Menot (2018) appear to have interpreted ‘slender’ as meaning less stout, noting that *B. mariaae* differs from the other three species in having notochaetae that are stouter, although always narrower, than the neurochaetae. Murray et al. (2025), by contrast, appear to have interpreted the character in terms of the relative thickness of the notochaetae. Based on the scaled drawings and species descriptions, we interpret Pettibone’s use of ‘slender’ to refer to width rather than robustness, that is, the notochaetae are narrower than the distally broadened and flattened neurochaetae. Taking the present study into account, the notochaetae appear to be consistently stouter across species, whereas the neurochaetae are broader distally but more fragile. This shows that even a long-used character can be applied inconsistently.

A few characters appear more promising for distinguishing species. The buccal organ of polynoids is generally described as an axial, muscular, eversible pharynx with terminal papillae and two pairs of dorsoventral jaws (Hutchings 2000; Hourdez 2022; Rouse et al. 2022). In the dissected pharynx of the new species, we observed a sac-like appendage at the distal end (Fig. 3I, J) which, when everted, superficially resembles the buccal bulb of *Aphrodita aculeata* Linnaeus, 1758, as illustrated in the figure redrawn by Hourdez et al. (2022). The same figure of buccal organ was labelled as the mouth in Rouse (2000). As far as we are aware, a comparable structure has not previously been reported at the distal end of the pharynx in any described species of *Bathyeliasona*. We include the buccal bulb in the diagnosis as a character observed in this species, while recognising that its broader taxonomic significance across the genus remains to be confirmed. Since the pharynx was not necessarily dissected in previous descriptions, and comparative material was not re-examined here, its apparent absence in congeners cannot be assumed. We therefore document this character with clear figures to flag it for future workers to examine and either validate or dismiss its diagnostic value. The shape of the pygidial lobes appears to be another useful character for distinguishing the new species from its congeners (Fig. 3H; Table 2), consistent with the taxonomic value of pygidial morphology at the species level noted previously by Bonifácio and Menot (2018).

Taken together, these observations suggest that the main challenge for species-level taxonomy in *Bathyeliasona* is not the absence of useful characters, but the difficulty of evaluating them consistently in the available material. Many promising characters, including nuchal organs, elytra, and delicate neurochaetae, are easily lost, damaged, or obscured during conventional trawling and dredging. Even so, *B. oceanxplorer* sp. nov. is clearly distinguished from its congeners by a combination of assessable morphological characters. The near-complete specimen also allowed the elytral morphology to be described for the first time in *Bathyeliasona*. The intact condition of this new species therefore highlights the value of targeted ROV sampling for observing and recovering large, delicate deep-sea annelids. As the present description is based on a single specimen, intraspecific variation could not be assessed, and additional material will be valuable for confirming the consistency of these characters. Improved ROV collections of fresh material will therefore help resolve which characters are consistently diagnostic within *Bathyeliasona* and clarify the diversity and distribution of the genus in poorly sampled regions such as the eastern Indian Ocean.

## Supplementary Material

XML Treatment for Bathyeliasona oceanxplorer

## Additional information

### Artificial Intelligence (AI) use

The authors accept full responsibility for the content of the manuscript, including the disclosure of any use of AI.

No AI tools were used in the preparation of this manuscript.

### Author ORCIDs

C.H. Chuar https://orcid.org/0000-0001-8423-440X

### Data availability

All of the data that support the findings of this study are available in the main text or Supplementary Information.

## Supplementary materials

Supplementary material 1 ROV clip https://doi.org/10.3897/zookeys.1293.208455.suppl1 Data type mov https://binary.pensoft.net/file/1790968This dataset is made available under the Open Database License (http://opendatacommons.org/licenses/odbl/1.0/). The Open Database License (ODbL) is a license agreement intended to allow users to freely share, modify, and use this Dataset while maintaining this same freedom for others, provided that the original source and author(s) are credited. Cheah Hoay Chuar, Iffah Iesa, Tashfia Raquib

## References

[B1] Bonifácio P, Menot L (2018) New genera and species from the Equatorial Pacific provide phylogenetic insights into deep-sea Polynoidae (Annelida). Zoological Journal of the Linnean Society 185: 555–635. 10.1093/zoolinnean/zly063

[B2] Britayev TA, Martin D (2006) Scale‐worms (Polychaeta, Polynoidae) associated with chaetopterid worms (Polychaeta, Chaetopteridae), with description of a new genus and species. Journal of Natural History 39: 4081–4099. 10.1080/00222930600556229

[B3] Capella-Gutiérrez S, Silla-Martínez JM, Gabaldón T (2009) trimAl: a tool for automated alignment trimming in large-scale phylogenetic analyses. Bioinformatics 25: 1972–1973. 10.1093/bioinformatics/btp348 PMC2712344 19505945

[B4] Carr CM, Hardy SM, Brown TM, Macdonald TA, Hebert PDN (2011) A tri-oceanic perspective: DNA barcoding reveals geographic structure and cryptic diversity in Canadian polychaetes. PLOS ONE 6: e22232. 10.1371/journal.pone.0022232 PMC3136506 21829451

[B5] Chernomor O, von Haeseler A, Minh BQ (2016) Terrace aware data structure for phylogenomic inference from supermatrices. Systematic Biology 65: 997–1008. 10.1093/sysbio/syw037 PMC5066062 27121966

[B6] Chuar CH, Hadiyanto H, Lee Y-L (2021) Annotated checklist of polychaetes from deeper waters of the Sunda Strait and eastern Indian Ocean off southwest Java, Indonesia. Raffles Bulletin of Zoology [Supplement No.] 36: 47–77. 10.26107/RBZ-2021-0030

[B7] Chuar CH, Hookabe N, Fujiwara Y, Iesa I, Kobayashi I, Zhang DS, Jimi N (2026) A new genus and two new species of asteroid-associated Polynoidae (Annelida: Polychaeta: Macellicephalinae) from the deep Indo-West Pacific. Raffles Bulletin of Zoology 74: 660–679.

[B8] Desbruyères D, Laubier L (1988) Exploitation d’une source de matière organique concentrée dans l’océan profond: intervention d’une annélide polychète nouvelle. Compte Rendu Hebdomaidaire des Séances de l’Académie des Sciences 307: 329–336.

[B9] Fauvel P (1913) Quatrième note préliminaire sur les polychètes provenant des campagnes de l’Hirondelle et de la Princesse-Alice, ou déposées dans le Musée Océanographique de Monaco. Bulletin de l’Institut Océanographique de Monaco 270: 1–80.

[B10] Flaxman B, Kupriyanova EK (2024) New species of *Laetmonice* (Aphroditidae, Annelida) from bathyal and abyssal depths around Australia. Records of the Australian Museum 76: 195–210. 10.3853/j.2201-4349.76.2024.1900

[B11] Gonzalez BC, Martínez A, Jamieson AJ (2025) Scale worm diversity in abyssal and hadal environments (Aphroditiformia, Annelida). Deep Sea Research Part I: Oceanographic Research Papers 220: 104490. 10.1016/j.dsr.2025.104490

[B12] Guindon S, Dufayard JF, Lefort V, Anisimova M, Hordijk W, Gascuel O (2010) New algorithms and methods to estimate maximum-likelihood phylogenies: assessing the performance of PhyML 3.0. Systematic Biology 59: 307–321. 10.1093/sysbio/syq010 20525638

[B13] Hartman O (1967) Polychaetous annelids collected by the USNS Eltanin and Staten Island cruises, chiefly from Antarctic Seas. Allan Hancock Monographs in Marine Biology 2: 1–387.

[B14] Hartmann-Schröder G (1971) Annelida, Borstenwürmer, Polychaeta. In: Dahl M, Peus F (Eds) Die Tierwelt Deutschlands und der Angrenzenden Meeresteile nach ihren Merkmalen und nach ihrer Lebensweise. Gustav Fischer Verlag, Jena, 1–594. 10.1086/407180

[B15] Hartmann-Schröder G (1975) Polychaeten der Iberischen tiefsee, gesammelt auf der 3. Reise der Meteor im Jahre 1966. Mitteilungen aus dem Hamburgischen Zoologischen Museum und Institut 72: 47–73.

[B16] Hatch AS, Liew H, Hourdez S, Rouse GW (2020) Hungry scale worms: Phylogenetics of *Peinaleopolynoe* (Polynoidae, Annelida), with four new species. Zookeys 932: 27–74. 10.3897/zookeys.932.48532 PMC7237507 32476973

[B17] Hoang DT, Chernomor O, von Haeseler A, Minh BQ, Vinh LS (2018) UFBoot2: Improving the ultrafast bootstrap approximation. Molecular Biology and Evolution 35: 518–522. 10.1093/molbev/msx281 PMC5850222 29077904

[B18] Hourdez S (2022) 7.13.1.4 Polynoidae Kinberg, 1856. In: Purschke G, Böggemann M, Westheide W (Eds) Volume 4 Pleistoannelida, Errantia II. De Gruyter, Berlin, Boston, 93–113. 10.1515/9783110647167-005

[B19] Hourdez S, Gonzalez B, Eibye-Jacobsen D (2022) 7.13.1 Aphroditiformia. In: Purschke G, Böggemann M, Westheide W (Eds) Volume 4 Pleistoannelida, Errantia II. De Gruyter, Berlin, Boston, 65–74. 10.1515/9783110647167-003

[B20] Hutchings PA (2000) Family Polynoidae. In: Beesley PL, Ross GJB, Glasby CJ (Eds) Polychaetes & Allies: The Southern Synthesis Fauna of Australia Vol 4A Polychaeta, Myzostomida, Pogonophora, Echiura, Sipuncula. CSIRO Publishing, Melbourne, 203–209.

[B21] Kalyaanamoorthy S, Minh BQ, Wong TKF, von Haeseler A, Jermiin LS (2017) ModelFinder: fast model selection for accurate phylogenetic estimates. Nature Methods 14: 587–589. 10.1038/nmeth.4285 PMC5453245 28481363

[B22] Katoh K, Standley DM (2013) MAFFT multiple sequence alignment software version 7: improvements in performance and usability. Molecular Biology and Evolution 30: 772–780. 10.1093/molbev/mst010 PMC3603318 23329690

[B23] Kinberg JGH (1856) Nya slägten och arter af Annelider. Öfversigt af Kongl Vetenskaps-Akademiens Förhandlingar 12: 381–388.

[B24] Kirkegaard JB (1956) Benthic Polychaeta from depths exceeding 6000 meters. Galathea Report 2: 63–78.

[B25] Kirkegaard JB (1995) Bathyal and abyssal polychaetes (errant species). Galathea Report 17: 7–56.

[B26] Kumar S, Stecher G, Suleski M, Sanderford M, Sharma S, Tamura K (2024) MEGA12: Molecular Evolutionary Genetic Analysis Version 12 for adaptive and green computing. Molecular Biology and Evolution 41: 1–9. 10.1093/molbev/msae263 PMC11683415 39708372

[B27] Kupriyanova EK, Haddad A, Gurgel R, Glasby CJ, Hutchings PA, Murray A, Paxton H, Wilson RS (2024) Catalogue of the Annelida collected by the IN2021_V04 and IN2022_V08 expeditions to the Australian Christmas Island and Cocos (Keeling) Islands Territories. Museum Victoria Science Reports 30: 1–71. 10.24199/j.mvsr.2024.30

[B28] Kupriyanova EK, Criscione F, Daffe G, Hutchings P, Lavesque N, Murray A, Paxton H, Budaeva N (2026) New species of Annelida of the Australian Indian Ocean Territory (Christmas Island and Cocos (Keeling) Islands) seamounts. Records of the Australian Museum 78: 139–186. 10.3853/j.2201-4349.78.2026.3013

[B29] Lê HLV, Lecointre G, Perasso R (1993) A 28S rRNA based phylogeny of the Gnathostomes: first steps in the analysis of conflict and congruences with morphologically based cladograms. Molecular Phylogenetics and Evolution 2: 31–51. 10.1006/mpev.1993.1005 8081546

[B30] Letunic I, Bork P (2024) Interactive Tree of Life (iTOL) v6: recent updates to the phylogenetic tree display and annotation tool. Nucleic Acids Reseach 52: W78–W82. 10.1093/nar/gkae268 PMC11223838 38613393

[B31] Levenstein RY (1961) Polychaeta from deep water in the Bering Sea. Trudy Instituta Okeanologia, Akademia Nauk SSSR 46: 147–178.

[B32] Levinsen GMR (1886) Kara-Havets Ledorme (Annulata). In: Lütken CF (Ed.) Djimphna-Togtets Zoologiske-botaniske Udbytte. J. Hagerup, Köbenhaven, 288–303.

[B33] Linnaeus C (1758) Systema Naturae per regna tria naturae, secundum classes, ordines, genera, species, cum characteribus, differentiis, synonymis, locis. Laurentii Salvii, Holmiae [Stockholm], [iii] + 824 pp. 10.5962/bhl.title.542

[B34] Medlin L, Elwood HJ, Stickel S, Sogin ML (1988) The characterization of enzymatically amplified eukaryotic 16S-like rRNA-coding regions. Gene 71: 491–499. 10.1016/0378-1119(88)90066-2 3224833

[B35] Miller MA, Pfeiffer W, Schwartz T (2010) Creating the CIPRES Science Gateway for inference of large phylogenetic trees. Proceedings of the Gateway Computing Environments Workshop (GCE): 1–8. 10.1109/GCE.2010.5676129

[B36] Miura T, Desbruyères D (1995) Two new species of *Opisthotrochopodus* (Polychaeta: Polynoidae: Branchinotogluminae) from the Lau and North Fiji Bac-arc Basins, southwestern Pacific Ocean. Proceedings of the Biological Society of Washington 108: 583–595.

[B37] Miura T, Hashimoto J (1991) Two new branchiate scale-worms (Polynoidae: Polychaeta) from the hydrothermal vent of the Okinawa Trough and the volcanic seamount off Chichijima Island. Proceedings of the Biological Society of Washington 104: 166–174.

[B38] Murray A, Burghardt I, Gunton LM, Nizar NM, Nikolic MC, Wilson RS (2025) Polynoidae (Annelida) from bathyal and abyssal depths in southern and eastern Australia. Records of the Australian Museum 77: 193–269. 10.3853/j.2201-4349.77.2025.1904

[B39] Neal L, Barnich R, Wiklund H, Glover AG (2012) A new genus and species of Polynoidae (Annelida, Polychaeta) from Pine Island Bay, Amundsen Sea, Southern Ocean—a region of high taxonomic novelty. Zootaxa 3542: 80–88. 10.11646/zootaxa.3542.1.4

[B40] Neal L, Brasier MJ, Wiklund H (2018) Six new species of *Macellicephala* (Annelida: Polynoidae) from the Southern Ocean and south Atlantic with re-description of type species. Zootaxa 4455: 1–34. 10.11646/zootaxa.4455.1.1 30314219

[B41] Neal L, Wiklund H, Smith CR, Benn A, Kemp K, Dahlgren TG, Glover AG (2026) New species of *Macellicephala* McIntosh, 1885 (Annelida, Polynoidae), associated with the reef stage of a whale fall. Taxonomy 6: 30. 10.3390/taxonomy6020030

[B42] Norlinder E, Nygren A, Wiklund H, Pleijel F (2012) Phylogeny of scale-worms (Aphroditiformia, Annelida), assessed from 18SrRNA, 28SrRNA, 16SrRNA, mitochondrial cytochrome c oxidase subunit I (COI), and morphology. Molecular Phylogenetics and Evolution 65: 490–500. 10.1016/j.ympev.2012.07.002 22789762

[B43] Nygren A, Sundberg P (2003) Phylogeny and evolution of reproductive modes in Autolytinae (Syllidae, Annelida). Molecular Phylogenetics and Evolution 29: 235–249. 10.1016/S1055-7903(03)00095-2 13678679

[B44] O’Hara TD (2024) The IN2021_V04 and IN2022_V08 expeditions to the Australian Christmas Island and Cocos (Keeling) Islands Territories. Museum Victoria Science Reports 23: 1–5. 10.24199/j.mvsr.2024.23

[B45] Palumbi SR (1996) Nucleic acids II: The polymerase chain reaction. In: Hillis DM, Moritz C, Mable BK (Eds) Molecular Systematics. Sinauer Associates, Sunderland, Massachusetts USA, 205–247.

[B46] Pettibone MH (1976) Revision of the genus *Macellicephala* McIntosh and the subfamily Macellicephalinae Hartmann-Schröder (Polychaeta: Polynoidae). Smithsonian Contributions to Zoology 229: 1–71. 10.5479/si.00810282.229

[B47] Pettibone MH (1985) Additional branchiate scale-worms (Polychaeta: Polynoidae) from Galapagos hydrothermal vent and rift-area off western Mexico at 21°N. Proceedings of the Biological Society of Washington 98: 447–469.

[B48] Pettibone MH (1989) Polynoidae and Sigalionidae (Polychaeta) from the Guaymas Basin, with descriptions of two new species, and additional records from hydrothermal vents of the Galapagos Rift, 21°N, and seep-sites in the Gulf of Mexico (Florida and Louisiana). Proceedings of the Biological Society of Washington 102: 154–168.

[B49] Read G, Fauchald K [Eds] (2026) World Polychaeta Database. *Bathyeliasona* Pettibone, 1976. World Register of Marine Species. https://www.marinespecies.org/aphia.php?p=taxdetails&id=129480 [Accessed on 18 March 2026]

[B50] Rouse GW (2000) Class Polychaeta: Morphology & Physiology. In: Beesley PL, Ross GJB, Glasby CJ (Eds) Polychaetes & Allies: The Southern Synthesis Fauna of Australia Vol 4A Polychaeta, Myzostomida, Pogonophora, Echiura, Sipuncula. CSIRO Publishing, Melbourne, 15–41.

[B51] Rouse GW, Pleijel F, Tilic E (2022) Polynoidae Kinberg, 1856. In: Annelida. Oxford University Press, 48–50. 10.1093/oso/9780199692309.003.0010

[B52] Sayers EW, Cavanaugh M, Frisse L, Pruitt KD, Schneider VA, Underwood BA, Yankie L, Karsch-Mizrachi I (2025) GenBank 2025 update. Nucleic Acids Research 53: D56–D61. 10.1093/nar/gkae1114 PMC11701615 39558184

[B53] Shields MA, Glover AG, Wiklund H (2013) Polynoid polychaetes of the Mid-Atlantic Ridge and a new holothurian association. Marine Biology Research 9: 547–553. 10.1080/17451000.2012.749992

[B54] Sjölin E, Erséus C, Källersjö M (2005) Phylogeny of Tubificidae (Annelida, Clitellata) based on mitochondrial and nuclear sequence data. Molecular Phylogenetics and Evolution 35: 431–441. 10.1016/j.ympev.2004.12.018 15804413

[B55] United Nations (2025) Statement by the Secretary-General – the entry into force of the BBNJ agreement. https://www.un.org/sg/en/content/sg/statements/2025-09-19/statement-the-secretary-general-the-entry-force-of-the-bbnj-agreement [Accessed on 09 July 2026]

[B56] Uschakov PV (1971) On a new abyssal species of *Macellicephala* McIntosh (Polychaeta, Errantia) from the Aleutian Trench. Trudy Instituta Okeanologia, Akademia Nauk SSSR 92: 36–40.

[B57] Wong TKF, Ly-Trong N, Ren HY, Demotte P, Baños H, Roger AJ, Susko E, Bielow C, De Maio N, Goldman N, Hahn MW, Dos Reis M, Vinh LS, Huttley G, Lanfear R, Minh BQ (2026) IQ-TREE 3: phylogenomic inference software using complex evolutionary models. Molecular Biology and Evolution 43: 1–6. 10.1093/molbev/msag117 PMC13191116 42085559

[B58] Wu X, Zhan Z, Xu K (2019) Two new and two rarely known species of *Branchinotogluma* (Annelida: Polynoidae) from deep-sea hydrothermal vents of the Manus Back-Arc basin, with remarks on the diversity and biogeography of vent polynoids. Deep Sea Research Part I: Oceanographic Research Papers 149: 103051. 10.1016/j.dsr.2019.05.011

[B59] Zhang Y, Chen C, Qiu J-W (2018) Sexually dimorphic scale worms (Annelida: Polynoidae) from hydrothermal vents in the Okinawa Trough: two new species and two new sex morphs. Frontiers in Marine Science 5: 112. 10.3389/fmars.2018.00112

